# An analysis and projection of diabetes prevalence in East England region

**DOI:** 10.1186/s12889-026-26433-1

**Published:** 2026-02-03

**Authors:** Ying Xie, Nasreen Anjum, Barbara Pierscionek, Mahreen Kiran, Magdalena Partac, Yue Wang

**Affiliations:** 1 Faculty of Business and Management, Cranfield University, Cranfield, UK; 2 School of Computing, University of Portsmouth, Portsmouth, UK; 3 Faculty of Health Medicine and Social Care, Medical Technology Research Centre, Cambridge, UK; 4 Anglia Ruskin University, Chelmsford, UK; 5 Faculty of Business and Law, Anglia Ruskin University, Chelmsford, UK; 6 School of Public Management, LiaoNing University, LiaoNing, China

**Keywords:** Diabetes Mellitus, Socioeconomic and demographic factors, Healthcare service access, Index of Multiple Deprivation (IMD)

## Abstract

**Background:**

Diabetes prevalence continues to rise in England, placing increasing pressure on primary and specialist healthcare services. This study examined how demographic, socioeconomic, and healthcare access factors influence Diabetes Mellitus register size across six Integrated Care Systems (ICSs) in the East of England and generated scenario based projections of future diabetes burden and specialist workforce requirements.

**Methods:**

A longitudinal panel design was applied using annual data (2012–2021) for six ICSs. Descriptive trend analysis summarised changes in diabetes registers, GP practice numbers, GP list size, deprivation (IMD scores), and population density. The association between these factors and Diabetes Mellitus register size was quantified using a *fixed effects panel regression model*, selected through F-tests, Breusch–Pagan LM tests, and Hausman specification testing. Future diabetes registers (2023–2027) were estimated using a regression based deterministic projection framework integrating: (i) model based forecasting, (ii) four scenario models based on plausible changes in population growth, deprivation, and GP capacity, and (iii) linear trend extrapolation of endocrinology consultant workforce numbers.

**Results:**

Diabetes registers increased across all ICSs, with the region experiencing a 13% rise between 2012 and 2021. Regression findings showed that higher deprivation strongly predicted larger diabetes registers ($\beta = 2824.25$, $p < 0.001$), while increases in GP list size and GP practice numbers were also significant predictors. Under Scenario I, projected diabetes registers for 2023 ranged from 60,603 (Cambridgeshire and Peterborough) to 85,574 (Hertfordshire and West Essex). Scenario II, which incorporated greater increases in deprivation, produced larger projected registers across all ICSs, including 75,463 in Bedfordshire and 88,399 in Hertfordshire. Patient to consultant ratios were projected to increase in Bedfordshire and Mid and South Essex, suggesting potential specialist workforce shortages.

**Conclusion:**

Demographic growth, rising deprivation, and pressures in primary care are key drivers of the increasing diabetes burden in the East of England. Projection results indicate that several ICSs may face widening gaps between patient demand and specialist capacity. Strengthening consultant staffing, directing resources toward more deprived areas, and supporting primary care resilience will be essential to maintain equitable diabetes care in future years.

## Introduction

Diabetes mellitus is a chronic condition marked by persistently high blood glucose levels due to inadequate insulin production or impaired insulin use [1, 2]. It has become a major public health concern in the UK, with over 4.3 million people currently living with diabetes. In 2021–22, registrations increased by nearly 149,000, while more than 2.4 million individuals remain at high risk of developing type 2 diabetes (T2D). An estimated 850,000 people have undiagnosed T2D, bringing the total number affected to over five million nationwide [3]. These trends highlight the urgent need for effective population-level prevention, early detection, and service planning.

The development and progression of T2D are influenced by a complex interaction of genetic, environmental, lifestyle, and socioeconomic factors. Genetic susceptibility affects glucose metabolism and insulin sensitivity, with higher prevalence observed in certain ethnic groups, including South Asian populations [4, 5]. Lifestyle factors such as unhealthy diet, physical inactivity, and obesity remain key drivers of T2D incidence [6, 7]. In addition, broader structural determinants, including urbanisation, population growth, and economic development, shape exposure to these risk factors and contribute to rising prevalence [8, 9]. Socioeconomic disadvantage is consistently associated with higher diabetes burden, reflecting disparities in access to healthcare, healthy food environments, and opportunities for physical activity [10, 11].

Previous research has extensively examined diabetes prevalence and its determinants at global and national levels. Studies have demonstrated strong associations between diabetes prevalence and socioeconomic conditions, healthcare quality, and population characteristics across a range of settings [10–13]. Large-scale modelling studies and international surveillance reports have projected substantial future increases in diabetes prevalence worldwide, particularly in low- and middle-income countries [14–17]. In England, national surveys and modelling studies indicate continued growth in diabetes prevalence driven by demographic change and rising obesity rates [18, 19].

Despite this extensive evidence base, important gaps remain. Much of the existing literature focuses on national or international trends, offering limited insight into regional variation within England, where sociodemographic conditions, deprivation, and healthcare capacity differ substantially. In particular, there is a lack of empirical research examining diabetes prevalence at the Integrated Care System (ICS) level, where healthcare planning and resource allocation increasingly occur. Furthermore, relatively little attention has been given to linking diabetes prevalence with healthcare workforce capacity and future workforce requirements, despite diabetes being a chronic condition with predictable service needs.

To address these gaps, this study investigates the relationship between sociodemographic factors, access to primary healthcare services, and diabetes prevalence across the six ICS in the East of England. Specifically, we examine the impact of population density, area-level deprivation measured by the Index of Multiple Deprivation (IMD), number of GP practices, and GP list size on diabetes registration. In addition, we project future changes in these factors and assess their potential implications for healthcare workforce requirements. By providing regionally focused evidence, this study aims to support more effective public health planning, targeted interventions, and workforce strategy development within the ICS framework.

## Methods

### Study design

This study employed a longitudinal panel design to assess how demographic, socioeconomic, and healthcare access factors shape variation in the Diabetes Mellitus register over time. A longitudinal panel design enables repeated observation of the same geographical units, allowing the analysis to capture both temporal dynamics and within-region variation. The analysis was undertaken at the level of ICSs, which are statutory partnerships responsible for planning and delivering health services for defined populations in England [20]. The six ICSs included in this study are listed in Table 1.

**Table 1:** Integrated care systems (ICSs), UK

Region	Areas included in Each Particular ICS Region
Bedfordshire	Bedfordshire, Luton and Milton Keynes
Cambridgeshire	Cambridgeshire and Peterboroug
Hertfordshire	Hertfordshire and West Essex
MidEssex	Mid and South Essex
Norfolk	Norfolk and Waveney Health and Care Partnership
Suffolk	Suffolk and North East Essex

For each ICS, annual data for the period 2012–2021 were compiled into a balanced panel dataset. This structure enabled examination of how year-to-year changes in demographic conditions (population density), socioeconomic disadvantage (IMD scores), and healthcare access indicators (number of GP practices and GP list size) were associated with corresponding changes in the DM register. The panel framework strengthens inference by accounting for unobserved, time-invariant characteristics of each ICS while modelling the impact of evolving contextual factors on diabetes burden.

The analysis was conducted in three stages: (1) data collection, (2) data preprocessing, and (3) data analysis.

### Step 1: data collection

All datasets used in this study were obtained from publicly accessible UK government sources. No special permissions, licences, or data-sharing agreements were required.

The study population comprised all adults aged 17 years and above registered with a GP in the East of England. Diabetes status was defined using the Quality and Outcomes Framework (QOF) disease register for diabetes (indicator DM017), which includes individuals with a clinician-confirmed diagnosis of Type 1 or T2D recorded in primary care according to national diagnostic guidelines [21]. Patients appearing on the DM017 register in a given reporting year were treated as diabetes cases for that year.

Data collection covered four main domains: diabetes prevalence, primary care provision, demographic and socioeconomic characteristics, and specialist workforce capacity.

#### Diabetes prevalence data

Disease prevalence data by disease type, year, GP practice, administrative area, and register counts were obtained from the Quality and Outcomes Framework (QOF), a national reporting system in which all GP practices in England submit standardised annual counts of patients diagnosed with chronic conditions, including diabetes. QOF datasets are published annually by NHS Digital and include practice-level and geographically aggregated statistics. All QOF data used in this study were downloaded directly from the NHS Digital website for each reporting year, including historical extractions such as the 2012–2013 release [22] and the 2019–2020 release [23]. The QOF diabetes register served as the primary measure of diabetes burden across the six ICSs in the East of England.

#### Primary care provision data

Information on primary care capacity was obtained from NHS Digital’s General Practice data releases, which include annual counts of active GP practices and GP list sizes for individuals aged 15 and above [24]. GP list size and practice counts are administrative indicators that measure how many people are registered for primary care and how many GP practices operate in each area. Both sources are comprehensive population-level datasets rather than samples.

#### Demographic and socioeconomic data

All demographic and deprivation datasets were accessed through the ONS website [25]. Mid-year population estimates were used to derive population density measures for each ICS. Socioeconomic deprivation was captured using the IMD, a UK-wide statistical measure derived from seven domains including income, education, employment, health, and living environment. The index is available for all small geographic areas, enabling robust comparison across regions. IMD scores for Lower Layer Super Output Areas (LSOAs) were aggregated to ICS level using population-weighted averages to ensure consistency across regions and years.

#### Healthcare workforce data

Data on specialist workforce capacity were obtained from NHS Digital’s workforce statistics, a monthly publication containing detailed staff counts by job role, grade and specialty[24]. Specifically, the number of consultant endocrinologists was extracted for each ICS and each year. These data were used to compute annual patient to consultant ratios as an indicator of specialist workload and capacity pressures related to diabetes care.

*Generalisability of Data*: Because all the above datasets are administrative systems covering all GP practices and populations in England, they have near-complete national coverage and are widely used in public health and health-services research. This enhances the generalisability of the study, as findings reflect the entire resident population of the East of England rather than a selected sample. However, generalisability beyond England should be considered with caution, as healthcare systems, socioeconomic structures, and disease coding practices may differ internationally.

### Step 2: data preprocessing

Following data collection, all datasets were cleaned, harmonised, and aligned to ensure comparability across years and ICSs. Annual observations were compiled for each of the six ICSs for the period 2012–2021. A key methodological challenge arises from the fact that historical health data in England are reported at different geographical levels. Older datasets are often available at the Clinical Commissioning Group (CCG) level, while more recent health system organisation is based on ICSs. In addition, some datasets are reported at finer spatial scales, such as Lower Layer Super Output Areas (LSOAs), or at intermediate planning levels, such as Sustainability and Transformation Partnerships (STPs). To ensure consistency, official NHS mapping tables were used to convert data reported at CCG, LSOA, or STP levels into the current ICS boundaries. This conversion was carried out by aggregating GP-level or population-weighted data so that all disease indicators align with the six ICSs included in the study.

Missing values, inconsistent labels, and structural differences in reporting formats were reviewed and addressed using standard data cleaning procedures. Variables from different sources were merged using ICS identifiers and reporting year. Numerical variables were checked for consistency of units before being standardised where appropriate.

Once the data were harmonised spatially and temporally, population health profiles were constructed for each ICS and for the East of England region as a whole, using the following analytic variables:

- *Diabetes Register*: Total number of adults (17+) recorded on the QOF DM017 register.
- *Diabetes Prevalence*: Calculated as
  $$\begin{aligned} \frac{\text {Diabetes Register}}{\text {GP List Size (15+)}} \times 100. \end{aligned}$$
- *GP List Size*: Total number of registered patients aged 15 and above.
- *Number of GP Practices*: Count of active general practices within each ICS.
- *IMD Score*: Population-weighted mean IMD score for Lower Layer Super Output Areas (LSOAs) within each ICS.
- *Population Density*: Number of persons per square kilometre based on ONS mid-year population estimates.
- *Patient-to-Consultant Ratio*: Diabetes register divided by the number of consultant endocrinologists in the corresponding ICS and year.

After cleaning, harmonisation, and merging, the final balanced panel dataset contained 54 observations (6 ICSs $\times$ 9 years). Table 2 (and its continuation) provides the full set of descriptive data. This dataset is used in all subsequent analyses.

**Table 2:** Data at ICS and East England 2012-2021

Year	Region name	DM* register (17+)	DP**	GPLS*** 15+	No of Practices	Population Density	IMD Score
2012-13	EAST OF ENGLAND	270480	5.966667	5048913	747	381.3617	16.085
	Bedfordshire Luton and Milton Keynes STP	43940	6.3	737198	114	550.25	17.08
	Cambridgeshire and Peterborough STP	38535	5.5	713011	107	225.9	15.3
	Hertfordshire and West Essex STP	60969	5.2	1190799	168	542.64	12.51
	Mid and South Essex	55789	6.1	955618	193	571.06	16.11
	Norfolk and Waveney Health and Care Partnership (STP)	43270	6.7	659874	95	170.89	18.89
	Suffolk and North East Essex STP	27977	6	792413	70	227.43	16.62
2013-14	EAST OF ENGLAND	282943	6.061354	5162125	739	384.7683	16.085
	Bedfordshire Luton and Milton Keynes STP	46184	6.38	756226	113	557.71	17.08
	Cambridgeshire and Peterborough STP	40002	5.576862	730445	107	227.23	15.3
	Hertfordshire and West Essex STP	62925	5.434165	1218718	167	548.16	12.51
	Mid and South Essex STP	57807	6.197352	973921	190	574.9	16.11
	Norfolk and Waveney Health and Care Partnership (STP)	46568	6.509396	674694	94	171.91	18.89
	Suffolk and North East Essex STP	29457	6.270347	808121	68	228.7	16.62
2014-15	EAST OF ENGLAND	290889	6.248492	5216673	732	389.3	16.085
	Bedfordshire Luton and Milton Keynes STP	47805	6.5	768255	113	565.99	17.08
	Cambridgeshire and Peterborough STP	41669	5.68095	747853	107	229.58	15.3
	Hertfordshire and West Essex STP	64852	5.53	1227954	167	554.87	12.51
	Mid and South Essex STP	59349	6.31	976519	187	580.86	16.11
	Norfolk and Waveney Health and Care Partnership (STP)	46587	7.04	682104	91	173.16	18.89
	Suffolk and North East Essex STP	30627	6.43	813988	67	231.34	16.62
2015-16	EAST OF ENGLAND	302615	6.392704	5281857	716	393.2517	17.64833
	Bedfordshire Luton and Milton Keynes STP	49491	6.6	780263	112	574.38	18.37
	Cambridgeshire and Peterborough STP	43296	5.756222	763295	106	232.2	16.28
	Hertfordshire and West Essex STP	67551	5.66	1241131	164	560.18	13.05
	Mid and South Essex STP	61076	6.42	984509	180	584.43	17.63
	Norfolk and Waveney Health and Care Partnership (STP)	48702	7.29	689256	89	174.48	21.57
	Suffolk and North East Essex STP	32499	6.63	823403	65	233.84	18.99
2016-17	EAST OF ENGLAND	343069	6.332623	5343209	776	397.0017	17.64833
	Bedfordshire Luton and Milton Keynes STP	51094	6.524723	791374	110	582.02	18.37
	Cambridgeshire and Peterborough STP	44861	5.852776	777603	106	233.98	16.28
	Hertfordshire and West Essex STP	70444	5.749165	1253897	162	565.23	13.05
	Mid and South Essex STP	60716	6.904363	992905	114	589.34	17.63
	Norfolk and Waveney Health and Care Partnership (STP)	53223	6.502592	695316	104	175.83	21.57
	Suffolk and North East Essex STP	62731	6.462117	832114	180	235.61	18.99
2017-18	EAST OF ENGLAND	352089	6.492821	5396421	743	399.3783	17.64833
	Bedfordshire Luton and Milton Keynes STP	53051	6.755688	803300	105	584.87	18.37
	Cambridgeshire and Peterborough STP	46573	5.965345	796086	102	235.7	16.28
	Hertfordshire and West Essex STP	61061	7.15761	1265052	106	567.86	13.05
	Mid and South Essex STP	54158	6.573259	999501	102	592.99	17.63
	Norfolk and Waveney Health and Care Partnership (STP)	72794	5.897216	690824	157	177.1	21.57
	Suffolk and North East Essex STP	64452	6.607805	841658	171	237.75	18.99
2018-19	EAST OF ENGLAND	360790	6.675157	5449351	679	401.4983	17.64833
	Bedfordshire Luton and Milton Keynes STP	54588	6.938479	814370	99	588.56	18.37
	Cambridgeshire and Peterborough STP	46513	6.065779	806541	87	237.17	16.28
	Hertfordshire and West Essex STP	75876	6.090862	1277787	144	570.1	13.05
	Mid and South Essex STP	63813	6.796672	1006886	150	596.18	17.63
	Norfolk and Waveney Health and Care Partnership (STP)	65294	7.475362	695824	105	178.08	21.57
	Suffolk and North East Essex STP	54706	6.683788	847943	94	238.9	18.99
2019-20	EAST OF ENGLAND	380754	6.84	5518927	681	403.8683	17.64667
	Bedfordshire Luton and Milton Keynes STP	57514	7.1	828999	99	592.63	17.95
	Cambridgeshire and Peterborough STP	50387	6.23	823733	87	238.13	16.7
	Hertfordshire and West Essex STP	78300	6.21	1291266	144	573.25	13.31
	Mid and South Essex STP	68719	6.94	1015639	151	600.14	16.98
	Norfolk and Waveney Health and Care Partnership (STP)	67405	7.62	700448	105	178.85	21.7
	Suffolk and North East Essex STP	58429	6.94	858842	95	240.21	19.24
2020-21	EAST OF ENGLAND	386205	6.893333	5767059	665	406.0956	17.64667
	Bedfordshire Luton and Milton Keynes STP	58933	7.17	838961	96	597.9638	17.95
	Cambridgeshire and Peterborough STP	51091	6.27	829487	85	239.28	16.7
	Hertfordshire and West Essex STP	78716	6.22	1302249	135	576.47	13.31
	Mid and South Essex STP	69374	6.99	1020453	150	602.02	16.98
	Norfolk and Waveney Health and Care Partnership (STP)	67891	7.61	909149	105	179.9	21.7
	Suffolk and North East Essex STP	60200	7.1	866760	94	240.94	19.24

In this Table, DM* refers to Diabetes Millennium, DP** to Diabetes prevalence, and GPLS*** to GP List size

### Step 3: data analysis

The analytical framework consisted of four components: descriptive analysis, econometric modelling, trend analysis of specialist workforce capacity, and scenario-based projection modelling. Each component addressed a different aspect of regional variation and future demand for diabetes services.

#### Descriptive analysis

Descriptive statistics were used to summarise temporal patterns in diabetes register counts, diabetes prevalence, GP list size, and demographic and deprivation indicators across the six ICSs.

**Fig. 1:**
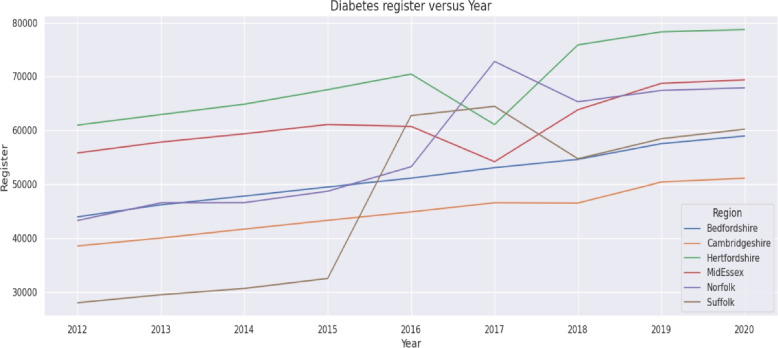
Diabetes register versus year in the six ICSs

Year-on-year percentage changes were then calculated for each indicator, with results presented in Table 3 (Diabetes Mellitus Register), Table 4 (Diabetes Mellitus Prevalence), and Table 5 (GP list size). Trends were visualised using time-series plots, including the diabetes register trajectory for each ICS (Fig. 1).

**Table 3:** Diabetes Mellitus Register (17+) - Year on Year (%) change

Region	2012-13	2013-14	Y/Y (%)	2014-15	Y/Y (%)	2015-16	Y/Y (%)	2016-17	Y/Y (%)
East of England	270,480	282,943	5%	290,889	3%	302,615	4%	343,069	13%
Bedfordshire Luton and Milton Keynes	43,940	46,184	5%	47,805	4%	49,491	4%	51,094	3%
Cambridgeshire and Peterborough	38,535	40,002	4%	41,669	4%	43,296	4%	44,861	4%
Hertfordshire and West Essex	60,969	62,925	3%	64,852	3%	67,551	4%	70,444	4%
Mid and South Essex STP	55,789	57,807	4%	59,349	3%	61,076	3%	60,716	-1%
Norfolk and Waveney Health and carePartnership (STP)	43,270	46,568	8%	46,587	0%	48,702	5%	53,223	9%
Suffolk and North East Essex STP	27,977	29,457	5%	30,627	4%	32,499	6%	62,731	93%

Region	2017-18	Y/Y (%)	2018-19	Y/Y (%)	2019-20	Y/Y (%)	2020-21	Y/Y (%)	Overall change (%)
East of England	352,089	3%	360,790	2%	380,754	6%	386,205	1%	43%
Bedfordshire Luton and Milton Keynes	53,051	4%	54,588	3%	57,514	5%	58,933	2%	34%
Cambridgeshire and Peterborough	46,573	4%	46,513	0%	50,387	8%	51,091	1%	33%
Hertfordshire and West Essex	61,061	-13%	75,876	24%	78,300	3%	78,716	1%	29%
Mid and South Essex STP	54,158	-11%	63,813	18%	68,719	8%	69,374	1%	24%
Norfolk and Waveney Health and care Partnership (STP)	72,794	37%	65,294	-10%	67,405	3%	67,891	1%	57%
Suffolk and North East Essex STP	64,452	3%	54,706	-15%	58,429	7%	60,200	3%	115%

**Table 4:** Diabetes Mellitus Prevalence - Year on Year (%) change

Region	2012-13	2013-14	Y/Y (%)	2014-15	Y/Y (%)	2015-16	Y/Y (%)	2016-17	Y/Y (%)
East of England	5.97	6.06	2%	6.25	3%	6.39	2%	6.33	-1%
Bedfordshire Luton and Milton Keynes STP	6.3	6.38	1%	6.5	2%	6.6	2%	6.52	-1%
Cambridgeshire and Peterborough STP	5.5	5.58	1%	5.68	2%	5.76	1%	5.85	2%
Hertfordshire and West Essex STP	5.2	5.43	5%	5.53	2%	5.66	2%	5.75	2%
Mid and South Essex STP	6.1	6.197	2%	6.31	2%	6.42	2%	6.9	8%
Norfolk and Waveney Health and Care Partnership (STP)	6.7	6.51	-3%	7.04	8%	7.29	4%	6.5	-11%
Suffolk and North East Essex STP	6	6.27	5%	6.43	3%	6.63	3%	6.46	-3%

Region	2017-18	Y/Y (%)	2018-19	Y/Y (%)	2019-20	Y/Y (%)	2020-21	Y/Y (%)	Overall change (%)
East of England	6.49	3%	6.68	3%	6.84	2%	6.89	1%	16%
Bedfordshire Luton and Milton Keynes STP	6.76	4%	6.94	3%	7.1	2%	7.17	1%	14%
Cambridgeshire and Peterborough STP	5.97	2%	6.07	2%	6.23	3%	6.27	1%	14%
Hertfordshire and West Essex STP	7.16	24%	6.09	-15%	6.21	2%	6.22	0%	20%
Mid and South Essex STP	6.57	-5%	6.8	3%	6.94	2%	6.99	1%	15%
Norfolk and Waveney Health and Care Partnership (STP)	5.9	-9%	7.48	27%	7.62	2%	7.61	0%	14%
Suffolk and North East Essex STP	6.61	2%	6.68	1%	6.94	4%	7.1	2%	18%

**Table 5:** GP list size (15+) - Year on Year changes (%)

Region	2012-13	2013-14	Y/Y (%)	2014-15	Y/Y (%)	2015-16	Y/Y (%)	2016-17	Y/Y (%)
East of England	5,048,913	5,162,125	2%	5,216,673	1%	5,281,857	1%	5,343,209	1%
Bedfordshire Luton and Milton Keynes STP	737,198	756,226	3%	768,255	2%	780,263	2%	791,374	1%
Cambridgeshire and Peterborough STP	713,011	730,445	2%	747,853	2%	763,295	2%	777,603	2%
Hertfordshire and West Essex STP	1,190,799	1,218,718	2%	1,227,954	1%	1,241,131	1%	1,253,897	1%
Mid and South Essex STP	955,618	973,921	2%	976,519	0%	984,509	1%	992,905	1%
Norfolk and Waveney Health and Care Partnership (STP)	659,874	674,694	2%	682,104	1%	689,256	1%	695,316	1%
Suffolk and North East Essex STP	792,413	808,121	2%	813,988	1%	823,403	1%	832,114	1%

Region	2017-18	Y/Y (%)	2018-19	Y/Y (%)	2019-20	Y/Y (%)	2020-21	Y/Y (%)	Overall change (%)
East of England	5,396,421	1%	5,449,351	1%	5,518,927	1%	5,767,059	4%	14%
Bedfordshire Luton and Milton Keynes STP	803,300	2%	814,370	1%	828,999	2%	838,961	1%	14%
Cambridgeshire and Peterborough STP	796,086	2%	806,541	1%	823,733	2%	829,487	1%	16%
Hertfordshire and West Essex STP	1,265,052	1%	1,277,787	1%	1,291,266	1%	1,302,249	1%	9%
Mid and South Essex STP	999,501	1%	1,006,886	1%	1,015,639	1%	1,020,453	0%	7%
Norfolk and Waveney Health and Care Partnership (STP)	690,824	-1%	695,824	1%	700,448	1%	909,149	30%	38%
Suffolk and North East Essex STP	841,658	1%	847,943	1%	858,842	1%	866,760	1%	9%

Year-on-year percentage change was computed using the standard formula:

$$\begin{aligned} \%\Delta _{t} = \left( \frac{X_{t} - X_{t-1}}{X_{t-1}}\right) \times 100, \end{aligned}$$

where $X_t$ denotes the value of the indicator in year *t*. This ensured a consistent method for quantifying annual variation across all ICSs.

To aid comparison across ICSs, descriptive summaries were produced by presenting key indicators side by side, including diabetes register counts and prevalence, number of GP practices, GP list size, IMD scores, and population density. These indicators were not combined into a single metric; instead, they were reported individually to provide a clear descriptive profile of each ICS and allow comparison with regional averages.

#### Econometric modelling (linear regression analysis)

To quantify the association between demographic, socioeconomic, and healthcare access variables and the size of the Diabetes Mellitus register, we estimated a set of longitudinal (panel-data) linear regression models. The dependent variable was the annual Diabetes Mellitus register count for adults aged 17 years and above. The explanatory variables included the number of GP practices, GP list size (aged 15+), population density, and IMD score.

Three standard panel-data estimators were evaluated [26]:

- *Pooled Ordinary Least Squares (OLS) Regression Model* [27]: assumes no systematic differences across ICS regions.
- *Fixed Effects (FE) Model* [28]: controls for unobserved, time-invariant ICS characteristics (e.g., geographical features or persistent structural conditions).
- *Random Effects (RE) Model* [29]: assumes ICS-specific effects are random and uncorrelated with the predictors.

Model selection followed the standard three-step approach used in applied panel-data analysis. First, an F-test for individual effects was used to assess whether the FE model provided a significantly better fit than the Pooled OLS model [30]. Second, the Breusch–Pagan Lagrange Multiplier test evaluated whether the RE specification was preferable to Pooled OLS [31]. Finally, the Hausman specification test compared the FE and RE models to determine whether the RE assumptions were valid [32]. The results of these tests supported the FEs specification, which was therefore selected as the final model. The estimated coefficients from the preferred FE model are presented in Table 6.

**Table 6:** Regression models of diabetes panel data alpha=$$0.05^*$$, alpha=$$0.01^{**}$$, alpha=$$0.001^{***}$$

	Pooled OLS	Fixed effect model	Random effect model
Intercept	$$-66060^{***}$$	$$-167200^{***}$$	$$-108700^{***}$$
Practices	$$129.20^{***}$$	$$204.15^{***}$$	$$157.95^{***}$$
IMD	$$3108.88^{***}$$	$$2824.25^{***}$$	$$4349.62^{***}$$
GP list size	$$0.06^{***}$$	$$0.05^{***}$$	$$0.078^{***}$$
Population density	-0.18	$$416.64^{**}$$	2.13
Bedfordshire		$$-135200^{***}$$	
Cambridgeshire		$$8764.75^{**}$$	
Hertfordshire		$$-131400^{***}$$	
MidEssex		$$-149200^{***}$$	
Norfolk		$$32750^{***}$$	
F-test	33.67	48.3	29.84
DF	49	44	49
**R⌃2**	0.733	0.908	0.709
AIC	1107	1060	1080
$\boldsymbol{\sigma }^{\boldsymbol{2}}\_\boldsymbol{\epsilon }$**, **SEE			16870172.66
$\boldsymbol{\sigma }^{\boldsymbol{2}}\_\boldsymbol{\mu }$			26102643.69
$\boldsymbol{\theta }$			0.74
Effect test		F-test=$$16.74^{***}$$	LM-test=$$13.38^{***}$$

#### Trend analysis of specialist workforce

To understand pressures on diabetes services over time, trends in the number of endocrinology consultants were analysed for each ICS from 2013 to 2020 and extended to 2028 using linear extrapolation. Data were visualised in Fig. 5, alongside trends in GP workforce numbers (Fig. 6) and historical patient-to-consultant ratios (Fig. 7). This analysis provided contextual insight into whether the specialist workforce expanded proportionally to the rising diabetes burden. The results informed the subsequent projection modelling by highlighting areas where consultant supply has historically lagged demand.

#### Projection modelling

**Fig. 2:**
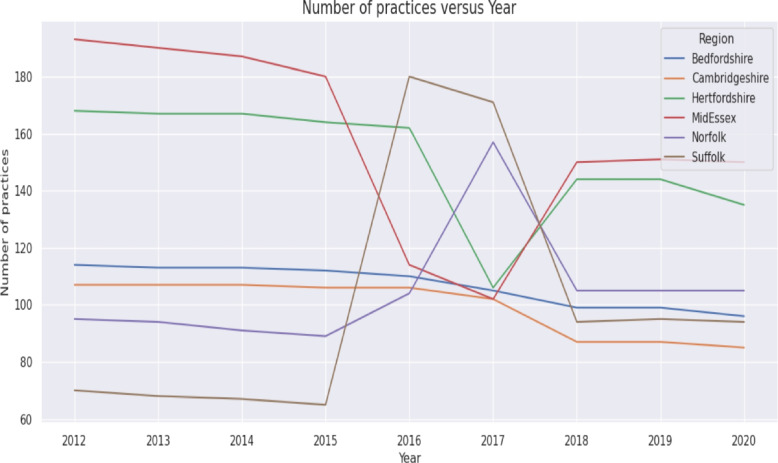
Number of practices versus year

**Fig. 3:**
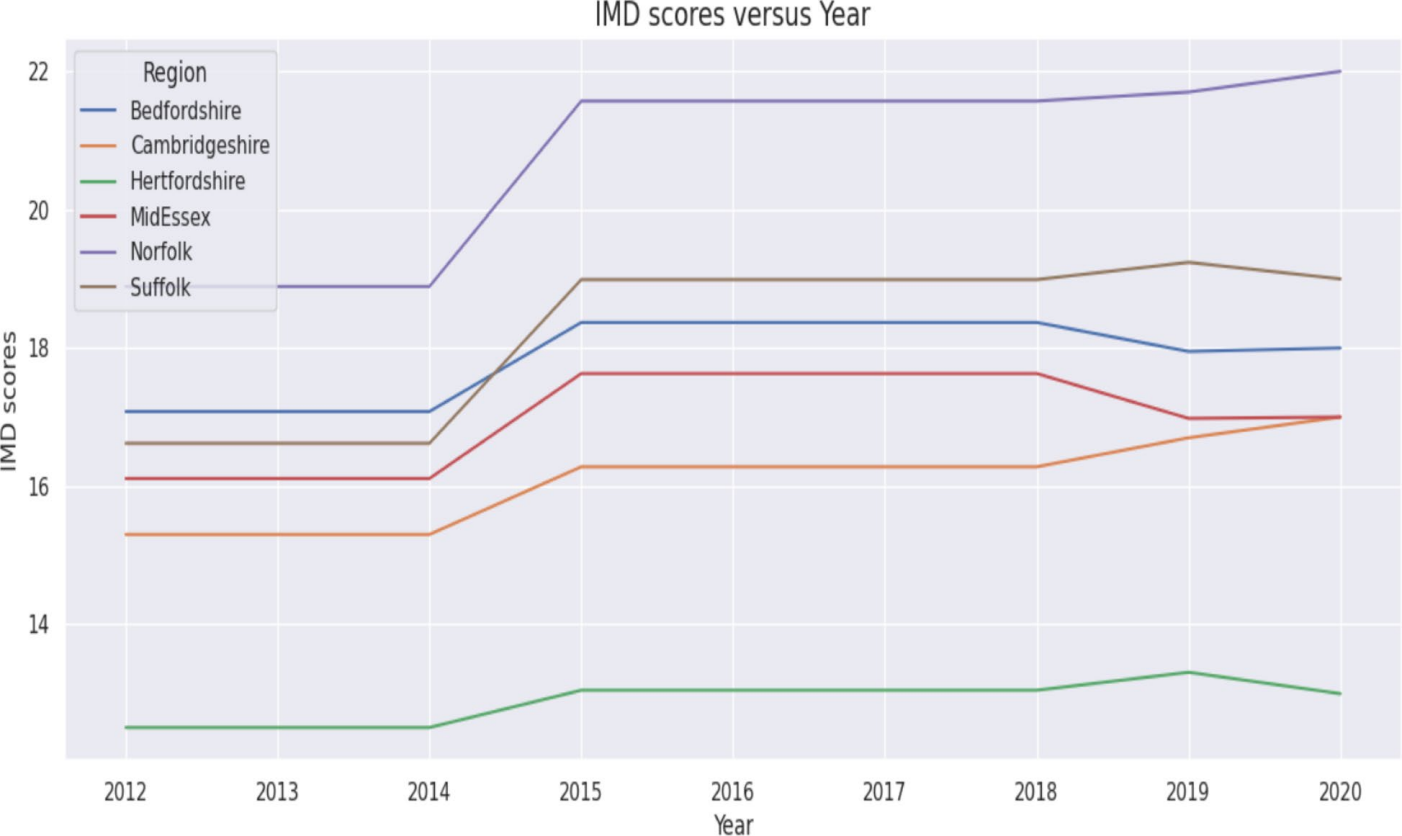
IMD scores versus year in the six ICSs

**Fig. 4:**
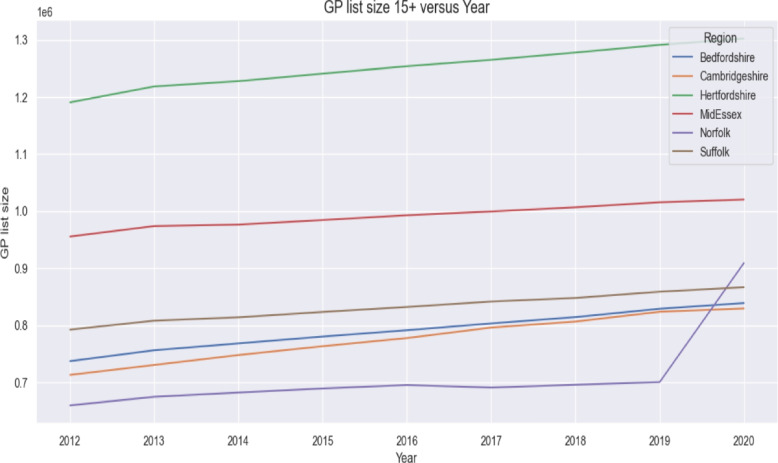
GP list size 15+ years old versus year in the six ICSs

Future diabetes registers and specialist workforce requirements were estimated using a regression based deterministic projection framework. This framework draws directly on the descriptive trends illustrated in Figs. 2, 4, and 3, as well as historical values summarised in Table 2. The projection analysis integrates three components: (i) model based forecasting using the estimated fixed effects regression model, (ii) deterministic scenario modelling based on historically observed changes, and (iii) linear trend extrapolation of the specialist workforce.

#### Model based projections using the fixed effects regression model

The projections were grounded in the final fixed effects model selected through the specification tests described earlier, with full coefficient estimates reported in Table 6. This model quantifies the association between the diabetes register and its determinants: number of GP practices, GP list size, IMD score, and population density.

Future values of the diabetes register were obtained by inserting projected covariate values into the estimated regression equation:

$$\begin{aligned} \widehat{DM\_register}_{it} & = \alpha _i + \beta _1 \, \text {Practices}_{it} + \beta _2 \, \text {IMD}_{it}\\ & \quad + \beta _3 \, \text {ListSize}_{it} + \beta _4 \, \text {PopDensity}_{it}, \end{aligned}$$

where $\alpha _i$ denotes ICS specific fixed effects and $\beta _k$ are estimated coefficients. This constitutes a structural regression forecasting approach in which projected input values (derived from historical trends shown in Figs. 4 and 3) are combined with model parameters to generate future estimates.

#### Deterministic scenario modelling

Because future trajectories of population growth, deprivation, and GP capacity may vary, four deterministic scenarios were constructed. These scenarios applied controlled incremental changes such as 1–3% annual increases in GP list size (informed by historical patterns in Fig. 4) and 1–2 point increases in IMD scores (based on historical variation shown in Fig. 3). Population density trends used to inform scenario increments were taken from historical data presented in Table 2.

Deterministic scenario modelling is widely used in health service planning to evaluate alternative plausible futures and quantify the sensitivity of projections to socioeconomic change.

#### Linear trend extrapolation for specialist workforce projections

Projected numbers of endocrinology consultants were generated using linear time trend extrapolation. Historical NHS workforce counts were used to fit a linear trend for each ICS, which was then projected forward for 2023–2028. The resulting projected consultant numbers for each ICS are summarised in Table 8. This extrapolation provides a conservative assumption regarding future specialist workforce availability.

#### Integration of projections

For each scenario, projected values for GP list size, IMD, and population density (informed by Table 7) were inserted into the fixed effects regression equation to generate predicted diabetes register counts for 2023–2027. These predicted register values were then combined with the projected consultant numbers (Table 8) to compute future patient to consultant ratios.

**Table 7:** Projected population in 2018-27 (source: ONS)

ICS	2018	2019	2020	2021	2022	2023	2024	2025	2026	2027
Bedfordshire, Luton and Milton Keynes	747794	752411	757065	762349	767564	773177	778325	783095	788144	793067
Cambridgeshire and Peterborough	723386	728188	732628	737340	742284	747480	752728	757840	763017	768657
Hertfordshire and West Essex	1186612	1191563	1196249	1202114	1208264	1214881	1221173	1227543	1233901	1240226
Mid and South Essex	968442	974463	980416	986973	993524	1000503	1007193	1013745	1020317	1027007
Norfolk and Waveney Health nd Care	674654	679990	685044	690480	696339	702457	708307	714163	720042	725759
Suffolk and North East Essex	811785	818018	823774	829815	835809	841889	847787	853792	859894	865857

**Table 8:** Projected number of endocrinology consultants in 2023-28

Year	Bedfordshire	Cambridgeshire	Hertfordshire	MidEssex	Norfolk	Suffolk
2023	17	47	50	28	40	37
2024	18	50	51	27	41	39
2025	16	51	50	27	42	40
2026	16	53	51	27	43	42
2027	16	54	52	27	44	43
2028	16	54	52	27	44	44

The final projected diabetes registers and corresponding workforce ratios for 2023 and 2024 are presented in Table 9. These results quantify the future burden under alternative demographic and socioeconomic assumptions.

**Table 9:** Projected patient register and patient consultant ratio in 2023 and 2024

Scenarios	ICS	Projected diabetes patient register	Projected diabetes patient consultant ratios
**Year 2023**			
**Scenario-I**	Bedfordshire Luton and Milton Keynes	72639	4272.86
	Cambridgeshire and Peterborough	60603	1289.43
	Hertfordshire and West Essex	85574	1711.49
	Mid and South Essex	77879	2781.40
	Norfolk and Waveney Health and Care Partnership	78958	1973.95
	Suffolk and North East Essex	60776	1642.60
**Scenario-II**	Bedfordshire Luton and Milton Keynes	75463	4439.00
	Cambridgeshire and Peterborough	63428	1349.52
	Hertfordshire and West Essex	88399	1767.97
	Mid and South Essex	80703	2882.27
	Norfolk and Waveney Health and Care Partnership	81782	2044.55
	Suffolk and North East Essex	63600	1718.93
**Year 2024**			
**Scenario-III**	Bedfordshire Luton and Milton Keynes	75550	4197.20
	Cambridgeshire and Peterborough	62014	1240.28
	Hertfordshire and West Essex	88625	1737.75
	Mid and South Essex	80898	2996.21
	Norfolk and Waveney Health and Care Partnership	80162	1955.18
	Suffolk and North East Essex	62214	1595.22
**Scenario-IV**	Bedfordshire Luton and Milton Keynes	78374	4354.11
	Cambridgeshire and Peterborough	64838	1296.76
	Hertfordshire and West Essex	91450	1793.13
	Mid and South Essex	83722	3100.81
	Norfolk and Waveney Health and Care Partnership	82987	2024.07
	Suffolk and North East Essex	65038	1667.64

This integrated approach provides a transparent framework for forecasting future diabetes burden and the associated workforce requirements.

#### Limitation of projection methodology

The projections generated in this study are based on a deterministic scenario–modelling approach in which fixed percentage changes in GP list size, IMD scores, and population density are inserted into the estimated fixed effects regression equation. Because the scenarios do not incorporate stochastic variation or probability distributions for future covariate values, the resulting estimates are point projections rather than statistical forecasts. Producing 95% confidence intervals would require a probabilistic framework (e.g., stochastic simulation, Bayesian forecasting, or Monte Carlo methods) that models uncertainty in both the regression parameters and future input variables. Such an approach is beyond the scope of this study and would require additional assumptions that may not be empirically supported. For this reason, confidence intervals are not presented, and the results should be interpreted as scenario–based projections rather than probabilistic estimates.

## Results

### Descriptive analysis results

Descriptive analysis provides a descriptive summary of temporal patterns in diabetes register counts, diabetes prevalence, and GP list size across the six ICSs in the East of England from 2012 to 2020. Figure 1 displays the annual Diabetes Mellitus register counts (17+) for each ICS from 2012 to 2020. All six ICSs show increases in register size over the study period, although the absolute levels and growth patterns differ between regions.

Table 3 summarises the numerical register values and the corresponding year-on-year percentage changes. The East of England increased from 270,480 cases in 2012 to 386,205 in 2020, an overall increase of 43%. All ICSs experienced positive overall growth, with annual percentage changes ranging from small incremental increases to sharp rises in specific years.

Table 4 reports annual diabetes prevalence and year-on-year percentage changes for each ICS and for the East of England as a whole. Regional prevalence rose from 5.97% in 2012 to 6.89% in 2020, representing a 16% overall increase. All ICSs demonstrated upward trends in prevalence across the period, although the magnitude of annual changes varied between regions.

Table 5 summarises year on year changes in GP list size for individuals aged fifteen and above across all ICSs. All regions demonstrated overall growth in GP registered populations between 2012 and 2021, although the rate of increase varied. The East of England as a whole recorded a fourteen percent increase in GP list size over the study period.

Together, Fig. 1, Tables 3, 4, and 5 provide a descriptive summary of temporal patterns in diabetes register counts, diabetes prevalence, and GP list size across the six ICSs in the East of England from 2012 to 2020.

### Linear regression analysis results

Linear regression results from the three types of models for Diabetes Mellitus are presented in Table 6.

The F statistic for the fixed effects model (16.74) exceeded the critical value of 5.02 at the 0.001 significance level, indicating that the fixed effects approach provides a significantly better fit than the pooled OLS model. The LM statistic for the random effects model (13.38) was greater than the chi square critical value of 10.83 at the 0.001 level, demonstrating that random effects are also significant. As both fixed and random effects were supported, a Hausman test was required to choose between the two model structures.

If the fixed effects model is selected, the Diabetes Mellitus register for each ICS can be expressed using the estimated coefficients:

$$\begin{aligned} & Register\ in\ Bedfordshire\\ & = (-167200 - 135200) + 204.15(Number\ of\ Practices)\\ & \quad + 2824.25(IMD) + 0.05(GP\ list\ size)\\ & \quad + 416.64(Population\ density) + error \end{aligned}$$

$$\begin{aligned} & Register\ in\ Cambridgeshire\\ & = (-167200 + 8764.75) + 204.15(Number\ of\ Practices)\\ & \quad + 2824.25(IMD) + 0.05(GP\ list\ size)\\ & \quad + 416.64(Population\ density) + error \end{aligned}$$

$$\begin{aligned} & Register\ in\ Hertfordshire\\ & = (-167200 - 131400) + 204.15(Number\ of\ Practices)\\ & \quad + 2824.25(IMD) + 0.05(GP\ list\ size)\\ & \quad + 416.64(Population\ density) + error \end{aligned}$$

$$\begin{aligned} & Register\ in\ MidEssex\\ & = (-167200 - 149200) + 204.15(Number\ of\ Practices)\\ & \quad + 2824.25(IMD) + 0.05(GP\ list\ size)\\ & \quad + 416.64(Population\ density) + error \end{aligned}$$

$$\begin{aligned} & Register\ in\ Norfolk\\ & = (-167200 + 32750) + 204.15(Number\ of\ Practices)\\ & \quad + 2824.25(IMD) + 0.05(GP\ list\ size)\\ & \quad + 416.64(Population\ density) + error \end{aligned}$$

$$\begin{aligned} & Register\ in\ Suffolk\\ & = -167200 + 204.15(Number\ of\ Practices)\\ & \quad + 2824.25(IMD) + 0.05(GP\ list\ size) \\ & \quad + 416.64(Population\ density) + error \end{aligned}$$

The marginal effects in the fixed effects model indicate:

1. For one unit increase in the Number of GP practices, the diabetes register is expected to increase by 204.15 units, holding all other variables constant.
2. For one unit increase in GP list size, the diabetes register is expected to increase by 0.05 unit, holding all other variables constant.
3. For one unit increase in IMD score, the diabetes register is expected to increase by 2824.25 units, holding all other variables constant.
4. For one unit increase in Population density, the diabetes register is expected to increase by 416.64 units, holding all other variables constant.

The regression coefficients presented in Table 6 quantify how variation in GP practice numbers, population deprivation, GP list size and population density contributes to annual changes in the Diabetes Mellitus register. To contextualise these estimates, Figs. 2, 3 and 4 illustrate the underlying temporal patterns of these explanatory variables over the study period. Figure 2 presents the annual number of GP practices across ICSs from 2012 to 2020, showing a general reduction over time with variation in the scale of decline across regions. Figure 3 displays IMD scores, illustrating year to year changes and upward shifts in several ICSs. Figure 4 shows GP list sizes for individuals aged 15 and above, which increased consistently across all ICSs during the study period. Together, these figures summarise the temporal patterns of the explanatory variables included in the regression models.

### Trend analysis of specialist workforce results

**Fig. 5:**
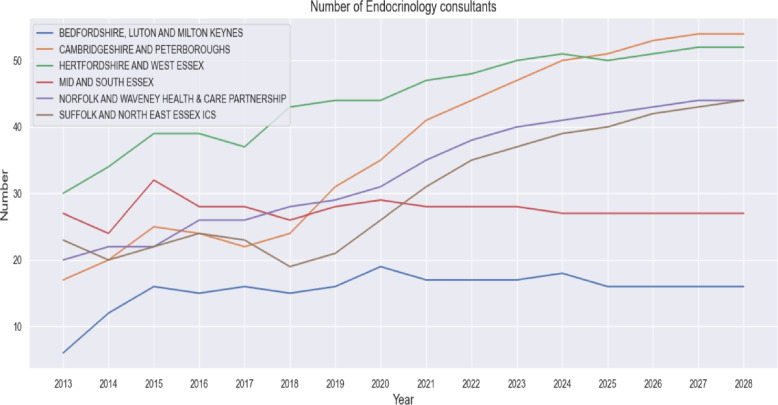
Number of Endocrinology consultants versus year in the six ICSs

Figure 5 presents the annual number of endocrinology consultants across the six ICSs. All regions show an overall increase from 2013 to 2028, although the rate and pattern of growth differ by area. Hertfordshire and West Essex consistently report the highest number of consultants, increasing from around 30 in 2013 to more than 50 in 2028. Cambridgeshire and Peterborough also display steady growth over time. In contrast, Bedfordshire, Luton and Milton Keynes maintains the lowest consultant numbers throughout the period, with only gradual increases after 2016. Mid and South Essex shows early fluctuations before stabilising, while Suffolk and Norfolk follow a more gradual upward trajectory.

**Fig. 6:**
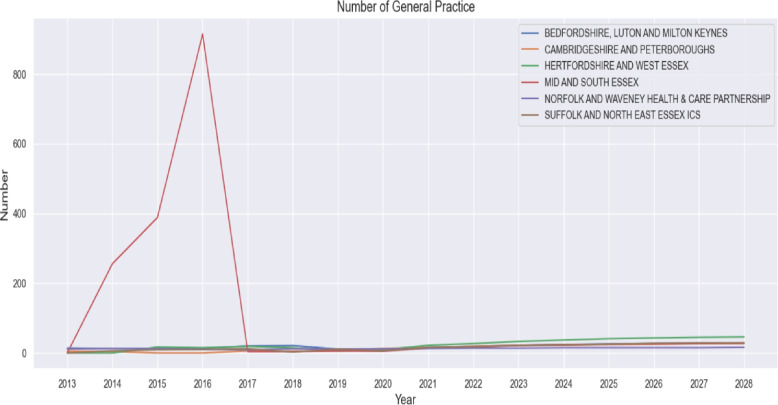
Number of GPs versus year in the six ICSs

Figure 6 illustrates the number of GPs from 2013 to 2028. With the exception of a substantial spike in 2016 in Mid and South Essex, which appears as an isolated data anomaly, GP numbers remain relatively stable across ICSs. Most regions show modest increases in GP numbers in the later years of the series, though the overall level of growth is considerably smaller than the increases observed in consultant numbers.

**Fig. 7:**
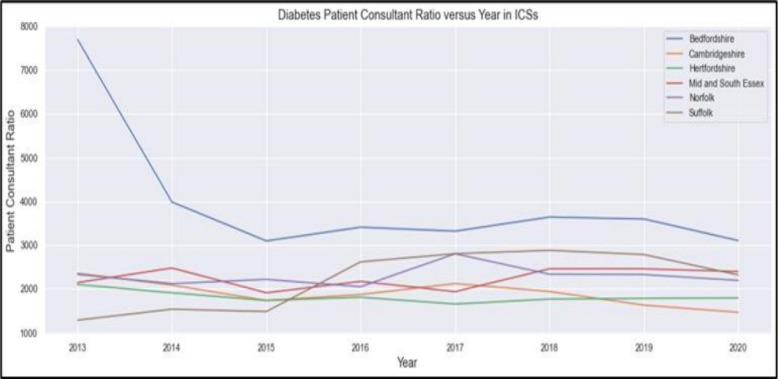
Diabetes Patient Consultant ratio versus year in the six ICSs

Figure 7 displays the diabetes patient to consultant ratio between 2013 and 2020. Ratios decrease over time in most ICSs, reflecting the combined effect of increasing consultant numbers and rising diabetes registers. Bedfordshire shows the highest ratios throughout the period but also the largest overall reduction, declining from around 7500 patients per consultant in 2013 to around 3000 by 2020. Other ICSs maintain lower and more stable ratios, typically falling between 1500 and 3000 patients per consultant during the later years of observation.

### Projections results

In Table 7, the projected population growth of 15+ years in 2018-27 is presented for each ICS. Comparing the projected population size of 15+ age groups in 2018-2020 with the actual GP list size 15+, we notice the projection underestimated the population sizes. For example, the actual GP list size in Bedfordshire, Luton and Milton Keynes in 2018 was 814,370, while the projected figure was 747,794 (9% less). The projected population size in Cambridgeshire was 728,188, 13% less than the actual GP list size 15+ of 823,733. Hence, when calculating the projected patient register, we make new projections based on the GP list size 15+ recorded in 2020. Considering the population increase in 2018, more realistic but conservative projections are made for population growth in 2023-2027 and are calculated as:

- Projection in 2023: Actual GP list size in 2020 * (1+2%)
- Projection in 2024: Actual GP list size in 2020 * (1+3%)
- Projection in 2025: Actual GP list size in 2020 * (1+4%)
- Projection in 2026: Actual GP list size in 2020 * (1+5%)
- Projection in 2027: Actual GP list size in 2020 * (1+6%)

Table 8 shows the corresponding projected speciality workforce growth for diabetes in each ICS. The diabetes patient registers in each ICS could be estimated for 2023-27 based on different scenarios. In these scenarios, we assume GP list size 15+ will be equal to the projected population size in 2023-27, leading to increased population density. Different increments in IMD scores are also modelled in the scenarios. Using the econometric model developed in Table 6, we could estimate the projected diabetes registers for each ICS under the different scenarios.

Using the projected population figures, workforce trajectories, and the regression-based forecasting framework, the next step was to apply the scenario assumptions to generate projected diabetes registers for each ICS. Four projection scenarios were examined: (i) Scenario I, based on a 2% increase in GP list size, a 1-point rise in IMD, and a 2% increase in population density; (ii) Scenario II, based on a 1% increase in GP list size, a 2-point rise in IMD, and a 1% increase in population density; (iii) Scenario III, based on a 3% increase in GP list size, a 1-point rise in IMD, and a 3% increase in population density; and (iv) Scenario IV, based on a 3% increase in GP list size, a 2-point rise in IMD, and a 3% increase in population density. These scenario-specific parameter values were substituted into the fixed effects regression model to generate projected diabetes register counts for each ICS. The results for each scenario and year are presented below, beginning with the projections for 2023.

#### Results for diabetes register projection in 2023

To evaluate how changes in demographic and socioeconomic conditions may shape future diabetes burden, two projection scenarios were constructed. These scenarios were not intended to exhaust all possible permutations, but rather to represent two distinct and practically meaningful trajectories based on observed historical trends and plausible future pressures. Developing a small number of contrasting but realistic scenarios aligns with standard practice in public health planning, where scenario modelling is used to establish lower and upper bounds for future service demand rather than produce exhaustive permutations. This approach promotes interpretability and avoids generating unnecessarily complex projections while still providing policymakers with meaningful decision-support [33].

- *Scenario I: 2% increment in GP list size, 1 point increment in IMD scores, 2% increase in population density*
  Scenario-I reflects a moderate change pathway, where GP list size, IMD scores, and population density increase at levels similar to recent historical patterns. This scenario represents a continuation of existing demographic growth without substantial socioeconomic deterioration.
  $$\begin{aligned} & Register\ in\ Bedfordshire\\ & = (-167200-135200) +204.15 * 96 + 2824.25\\ & \quad * (17.95 + 1) + 0.05 * 838961 * (1+0.02)\\ & \quad + 416.64 *598 * (1+0.02) + 5000 \end{aligned}$$
  $$\begin{aligned} & Register\ in\ Cambridgeshire\\ & =(-167200 + 8764.75) + 204.15 * 85 + 2824.25\\ & \quad *( 17.7+1) + 0.05 * 829487 *(1+0.02)\\ & \quad + 416.64 * 239 * (1+0.02) + 5000 \end{aligned}$$
  $$\begin{aligned} & Register\ in\ Hertfordshire\\ & = (-167200-131400) + 204.15 * 135 + 2824.25\\ & \quad * (13.31 + 1) + 0.05 * 1302249 * (1 + 0.02)\\ & \quad + 416.64 * 576 *(1 + 0.02) + 5000 \end{aligned}$$
  $$\begin{aligned} & Register\ in\ MidEssex\\ & = (-167200 - 149200) + 204.15 * 150 + 2824.25\\ & \quad * (16.98 + 1) + 0.05 * 1020453 * (1 + 0.02)\\ & \quad + 416.64 * 602 * (1 + 0.02) + 5000 \end{aligned}$$
  $$\begin{aligned} & Register\ in\ Norfolk\\ & = (-167200 + 32750) + 204.15 * 105 + 2824.25\\ & \quad * (21.7+ 1) + 0.05 *909149 *(1 + 0.02)\\ & \quad + 416.64 * 180 *(1 + 0.02) + 5000 \end{aligned}$$
  $$\begin{aligned} & Register\ in\ Suffolk\\ & = -167200 + 204.15 *94 + 2824.25 *(19.24 + 1)\\ & \quad + 0.05 *866760 *(1 + 0.02) + 416.64\\ & \quad * 241 *(1 + 0.02) + 5000 \end{aligned}$$
  Using the projected workforce growth information in Table 8, we could predict the patient consultant ratio of diabetes disease at the ICS level in 2023 (see Table 9).
- *Scenario II: 1% increment in GP list size, 2 points increment in IMD scores, 1% increase in population density*
  Scenario II assumes a higher socioeconomic stress trajectory, characterised by a smaller increase in GP list size (1%) but a larger deterioration in deprivation (2-point increase in IMD score) and a 1% rise in population density. Under this scenario, the projected diabetes registers are consistently higher across all ICSs compared with Scenario I (Table 9), highlighting the dominant influence of deprivation on future diabetes burden.
  For example, in Bedfordshire, Luton and Milton Keynes, the projected diabetes register increases from 72,639 in Scenario I to 75,463 in Scenario II, representing an additional 2,824 patients attributable primarily to higher deprivation. Similarly, Cambridgeshire and Peterborough shows an increase from 60,603 to 63,428 patients, while Hertfordshire and West Essex rises from 85,574 to 88,399. Comparable upward shifts are observed in Mid and South Essex (77,879 to 80,703), Norfolk and Waveney (78,958 to 81,782), and Suffolk and North East Essex (60,776 to 63,600).
  These results demonstrate that even modest increases in deprivation have a disproportionate effect on diabetes burden relative to changes in population size or GP list growth. Scenario II therefore represents a higher-pressure future in which worsening socioeconomic conditions accelerate demand for diabetes services, reinforcing the need for preventive and social policy interventions alongside healthcare capacity planning.

#### Results for diabetes register projection in 2024

The same two scenarios were considered for the projection in 2024, but the projected GP list size and population density are 3% more than the numbers in 2020, and IMD increments remain the same. The two scenarios are:

- *Scenario-III*: 3% increment in GP list size, 1 point increment in IMD scores, 3% increase in population density
- *Scenario-IV*: 3% increment in GP list size, 2 points increment in IMD scores, 3% increase in population density

In Cambridgeshire, Hertfordshire, Norfolk, and Suffolk, the estimated diabetes Patient Consultant ratios in both scenarios are consistent with the historical ratios in 2019 and 2020. We observed increments in the Patient Consultant ratios for both Bedfordshire and Mid and South Essex, indicating that more workforces may be required in these areas.

## Discussion

### Discussion of descriptive analysis

The descriptive results from this study show a steady increase in the burden of diagnosed diabetes across all six ICSs in the East of England from 2012 to 2020. As illustrated in Fig. 1, every ICS experienced a rising pattern in diabetes register counts, although the rate of increase varied between regions.

The year on year changes presented in Table 3 indicate that all ICSs experienced growth in their diabetes registers, but the size and timing of these increases differed. Certain ICSs such as Suffolk and Norfolk displayed notable fluctuations in specific years, whereas others such as Bedfordshire and Cambridgeshire followed more stable upward patterns. Between 2012 and 2020 the Diabetes Mellitus register has increased in the East of England by 43% from 270,480 to 386,205. The increase was observed acorss all ICSs in the region. The increase in the Diabetes Mellitus register (43%) in the East of England was higher than the increase in England over the same period (26%).

A similar pattern appears in Table 4, which shows that diabetes prevalence rose across the East of England, although the amount of increase differed between ICSs. Some areas experienced more rapid increases, while others showed slower and more steady growth. Between 2012 and 2020, there was an upward trend in diabetes prevalence in the East of England. The prevalence increased by 16%, rising from 5.97 to 6.89. This indicates a higher proportion of individuals in the population being affected by diabetes during this period. Unlike the register, the highest increase in the prevalence was recorded in Hertfordshire and West Essex ICS. Suffolk was the other ICS that registered an above-average increase, at 18%. The difference between the 115% increase in the Suffolk diabetes register and the 18% increase in prevalence can be explained by the rate of GP list size increase in the ICS. The GP list size increase (from 570,472 to 848,257) absorbed the increase in the number of patients registered with Diabetes Mellitus (from 27,977 to 60,200). All other ICSs in the area reported lower than the England average growth in prevalence (between 14-15% while England as a whole has a 16% increase). However, it is worth mentioning that over the entire period, East of England reported a lower prevalence than the average level in England (2012: 5.97 vs 6.1; 2020: 6.89 vs 7.08).

The patterns observed in Table 5 show that GP list sizes increased steadily across all ICSs throughout the study period, although the pace of this growth varied. Most ICSs recorded small but consistent annual increases of one to two percent, indicating gradual population expansion and a rising number of individuals registered with primary care services. Cambridgeshire and Bedfordshire were characterised by particularly stable and uniform growth, reflecting relatively predictable demographic change. In contrast, Norfolk and Waveney displayed a notable deviation from this pattern. While showing modest increases in earlier years, the ICS experienced a sharp rise in GP list size in the final year, resulting in the largest overall increase across the region. This sudden change suggests a substantial shift in the registered population, which may be linked to demographic movements, boundary adjustments, or changes in GP registration behaviour. Hertfordshire and West Essex showed steady growth but recorded one of the lowest overall percentage increases in list size, despite being one of the most populous ICSs. This relative stability contrasts with the larger increases observed in Suffolk and Cambridgeshire and may help explain some of the differences seen in diabetes prevalence trends across regions.

When interpreted alongside the diabetes register and prevalence results, these patterns highlight the importance of accounting for changes in the denominator population. Regions with rapid increases in GP list size did not always exhibit equivalent increases in diabetes prevalence, because population expansion can dilute prevalence even when case numbers are rising. Conversely, areas with slower list size growth showed clearer increases in prevalence, reflecting a tighter relationship between new diagnoses and population change.

Overall, these findings are consistent with national surveillance data indicating that diabetes prevalence in England has continued to rise in recent years. National statistics show that the number of people diagnosed with T2D and other forms of diabetes in England has increased over recent reporting periods, with the overall prevalence of diagnosed and undiagnosed T2D estimated at 7.8% in 2021 and rising trends in registered diabetes counts [34]. Furthermore, charity reports indicate that the total number of people living with diabetes in the UK is at an all-time high and continues to grow, placing increasing pressure on primary care and specialist services [35].

Taken together, these descriptive findings highlight three important themes. First, diabetes is increasing in every ICS in the East of England, although not at the same pace. Second, changes in population size and primary care configuration are likely to influence both the number of diagnoses recorded and the size of the denominator used to calculate prevalence. Third, ICSs with higher deprivation scores, including Bedfordshire, Norfolk, and Suffolk, are also areas where higher diabetes burden has been reported in other public health studies, supporting the evidence that socioeconomic disadvantage plays an important role in shaping regional patterns of chronic disease [36].

### Discussion of regression analysis

Key points derived from the regression models are:

- The model’s R${} ^2$ is 0.908 which shows that the proposed model explains 90.8
- All coefficients are statistically significant and positive meaning any increase in the determinant factors will result in an increase of the Diabetes Mellitus register.
- *Impact of Number of GP Surgeries*: When the descriptive patterns summarised in Discussion of descriptive analysis section are considered alongside the regression estimates reported in Linear regression analysis results section, a clearer picture emerges regarding how structural changes in primary care provision influence the diabetes register.
  1. Results show that increasing the number of GP surgeries in the area will result in an increase of the Diabetes Mellitus register. However, in the East of England, between 2012 and 2020, the number of GP surgeries has decreased by 82 practices which is associated with a decrease on the Diabetes register of 16,740 patients (see Table 2). Whilst in most ICSs the number of practices decreased, in both Norfolk and Suffolk the number of practices increased (by 10 and 24 respectively) thus contributing to a cumulative increase of 6,941 patients across the two ICS (see Fig. 2). This pattern of decreasing GP practices is consistent across England. Consequently, this trend suggests a potential decrease in the Diabetes Mellitus register due to the decreasing availability of GP surgeries in the future.
  2. Between 2012 and 2020, the number of GP practices in England has also recorded a downward trend however the rate of decrease (-18%) was higher than in East of England (-11%). Therefore, as the trend is consistent for both East of England and England, the number of GP practices is likely to continue to decrease which would result in a decrease in the Diabetes Mellitus register QOF201213 [22], QOF201314 [37], QOF201415 [38], QOF201516 [39], QOF201617 [40], QOF201718 [41], QOF201819 [42], QOF201920 [23].
  The observed relationship is supported by national evidence showing that despite rising patient numbers, the ratio of GPs to patients has declined in recent years, indicating increasing pressure on primary care and reduced access[43]. Therefore, the decline in the number of GP practices in both our region and across England [22, 23] is a serious concern. With fewer practices available to see patients, the ability to find and diagnose new cases of diabetes may be reduced. This could cause the official count of diabetes patients to appear lower, even if the actual number of people with the disease in the community is still rising.
- *Impact of Deprivation IMD Score*: A one-point increase in the IMD is associated with a rise of 2,824.25 patients on the diabetes register. Between 2012 and 2020, IMD in the region has increased by 1.56 which is associated with a 4409 increase in the Diabetes Mellitus register (See Table 2). Since IMD tends to increase across the region (Fig. 3), it is anticipated that the number of registered diabetes patients will continue to grow. This finding is consistent with evidence showing that socioeconomic deprivation is a strong and independent predictor of T2D risk and prevalence in England [18, 44, 45].
- *Impact of GP List Size*: An increase in the GP list size of 100 patients would result in an additional 5 people registering with Diabetes Mellitus (see Fig. 4 and Table 2). During the reporting period, the GP list size (15+) for Suffolk and North East Essex ICS has increased by 74,347 resulting in an overall Diabetes register increase of 3,717. Similarly, in Norfolk and Waveney Health and Care Partnership, the GP list size increased by 249,275 resulting in an increase in the Diabetes register of 12,464. These finding reflects the fundamental link between population size and disease burden within a primary care system. As the population registered with a practice grows whether from natural increase, aging, or inward migration, the absolute number of individuals with chronic conditions like diabetes is expected to rise proportionally, assuming a stable underlying prevalence [19]. In a system with a finite number of GP practices, such population growth directly increases list sizes, placing greater demand on existing primary care resources for chronic disease management [46].
- *Impact of Population Density*: An increase in population density by one person per square kilometer is associated with a 416.64 patient increase on the Diabetes Mellitus register. Between 2012 and 2020, East of England’s population density increased by an average of 24.71 people/km2 which is associated with an increase of the register of 10,305 patients. However, the increase was not uniform across the region. Bedfordshire reported the highest increase in population density of 47.71 people/km2 explaining a 19,879 increase in the diabetes register. Similarly, Hertfordshire and Mid and South Essex reported high increases in population density of 33.83 and 30.96 resulting in an increase of the register of 14,095 and 12,899 respectively. Norfolk reported the lowest increase in population density associated with a 3,753 increase in the diabetes register. The increase in population density reverses the decrease in the number of patients explained by the decrease in the number of GPs (See Table 2). In summary, our model identified population density as a significant, independent predictor of the diabetes register. This association aligns with extensive research on the health implications of urbanisation. Denser, more urbanized environments can influence diabetes risk through complex pathways. These often include lifestyle factors associated with urban living, such as diets higher in processed foods, more sedentary occupations, and environments that may discourage physical activity [47]. Furthermore, population growth in an area with a static or shrinking number of GP practices, as observed in our study, leads to larger practice list sizes and increased pressure on primary care services, potentially affecting both the detection and management of chronic diseases [46]. The regional variation we observed, with the greatest diabetes burden linked to the fastest-growing and densest ICSs like Bedfordshire, underscores the need for targeted resource planning and public health interventions in these high-growth urban and suburban areas.

### Discussion of specialist workforce trends

The trend analysis highlights several important patterns relevant to diabetes workforce planning in the East of England. First, the steady increase in endocrinology consultants across most ICSs (Fig. 5) indicates gradual strengthening of specialist capacity. This aligns with national policy directives, such as the NHS Long Term Plan, which emphasise expanding specialist roles to manage complex chronic conditions [48]. However, marked variation persists between regions. Bedfordshire consistently has the smallest specialist workforce, while Hertfordshire and West Essex maintain substantially higher numbers. Such geographical inequity in specialist distribution is a recognized challenge within the NHS, contributing to variation in care quality and patient access, particularly for long-term conditions like diabetes [49].

The patterns observed in GP numbers (Fig. 6) contrast with the specialist trends. GP numbers remain relatively stable, with only modest increases in later years. This limited growth in primary care capacity is of particular concern because GPs are the frontline for diabetes detection, monitoring, and management. A growing body of evidence links robust primary care provision to better chronic disease outcomes and reduced health inequalities[49]. The stagnation in GP numbers amidst a rising diabetes caseload risks overburdening existing practices, potentially compromising the continuity and quality of care[49].

The diabetes patient-to-consultant ratios (Fig. 7) provide an integrated view of specialist workload. Ratios have declined across most ICSs, suggesting some improvement. Nevertheless, Bedfordshire continues to carry a comparatively high workload burden. These persistently high ratios indicate a potential mismatch between service demand and specialist supply, which can lead to longer waiting times and suboptimal disease control for patients, as noted in other studies of specialist service pressures [50].

Taken together, these findings suggest that while specialist capacity is strengthening, growth is uneven and may not match the increasing diabetes burden. Effective workforce planning must therefore involve a dual strategy: sustained consultant expansion aligned with local disease prevalence, and a renewed focus on supporting and expanding the primary care workforce to create a sustainable, integrated model of diabetes care that addresses persistent regional disparities.

#### Discussion of projection findings

The projection analysis indicates that earlier population forecasts underestimated the size of the adult population across several ICSs, which has important implications for anticipating future demand for diabetes services. When updated population estimates based on the 2020 GP list size were used, the projected diabetes registers increased accordingly, reflecting the strong influence of population growth on service needs.

Across all scenarios, changes in GP list size, deprivation levels, and population density produced measurable shifts in the estimated diabetes registers. The higher register values observed in Scenario II and Scenario IV demonstrate that increases in IMD scores have a pronounced effect on projected diabetes burden. This finding aligns with established evidence linking deprivation to higher risk of chronic disease, reinforcing the need for a coordinated response that addresses both clinical and social determinants of health. The strong association between socioeconomic deprivation and diabetes risk is well documented, with national analyses showing that diabetes prevalence increases sharply across IMD quintiles [51]. These findings reinforce the need for coordinated approaches that address both clinical and social determinants of health, a core principle in the NHS Long Term Plan and population–health management frameworks [52].

Projected patient consultant ratios for 2023 and 2024 show broad stability in Cambridgeshire, Hertfordshire, Norfolk, and Suffolk, where future ratios remain close to those reported in 2019 and 2020. In contrast, Bedfordshire and Mid and South Essex demonstrate rising ratios across scenarios, suggesting that current workforce growth may not be sufficient to meet future needs. These patterns highlight the value of using predictive modelling to support strategic workforce planning and to target investment where pressures are likely to be greatest. Similar concerns have been highlighted in national workforce reports, which show persistent shortages in specialist medical staff, including endocrinology, and warn that demand is likely to outpace future workforce expansion without targeted intervention [48, 53]. These patterns highlight the value of predictive modelling for supporting strategic workforce planning and ensuring alignment between population need and specialist capacity.

Taken together, the projections illustrate how demographic change, deprivation, and variations in workforce capacity interact to shape future demand for specialist diabetes care. Ensuring adequate consultant supply, particularly in ICSs where patient consultant ratios are projected to rise, will be essential for maintaining equitable access to diabetes services across the region.

### Practical and theoretical implications

These findings highlight the need for a review of healthcare provision and the need for greater local intervention and authority. It is an ideal opportunity for the UK governments ICSs to effectively address and tackle these health problems as ICSs were established to take a regional and joint care approach. The intention is for ICSs to focus on health and care service improvement to reduce inequalities in health and to look at preventative measures. The joint up care vision brings together regional NHS Trusts, councils, voluntary sector and other local partners to co-create effective services that meet local needs. Within the ICSs, Integrated Care Boards (ICBs) are tasked with planning health service provision together with NHS providers. This noble aim, notwithstanding the existing issues with diabetes, require rapid action and a need to redress existing inequalities in both a speedy and sustained way. Diabetes is a chronic disease with predictable outcomes which means planning for improved healthcare and enhanced economic development for the long-term is possible. Diabetes, as reinforced from these findings is associate with deprivation and hence, again it is relatively easy to find the areas of higher incidence and prevalence. The difficulty with diabetes is that much of the treatment depends on the patient behaviour and compliance. Public health programmes have had limited effectiveness but should not be abandoned. Technology should and could be used to help with communicating the seriousness of the condition, the consequences of an unhealthy lifestyle and the need for patients to take greater responsibility for their health.

## Conclusion and future direction

This research utilised econometric modeling to explore the effects of diverse socio-demographic factors and access to healthcare service provision on the prevalence of Diabetes Mellitus across the six ICSs in the East of England. The factors considered included the number of GP practices, GP list size, population density, and IMD scores. Furthermore, the study anticipated future trends in the prevalence of Diabetes Mellitus and the associated consultant-to-patient ratios, taking into account the different levels of change in socio-demographic factors and access to healthcare service provision. This projection aims to offer insights into the anticipated workload for current endocrinology consultants and support future workforce planning efforts. The study overall highlights that increasing number of GP surgeries, increasing deprivation level, larger GP list sizes and higher population density are associated with an increase in the Diabetes Mellitus register. While there are some variations across different regions, the overall trends suggest a likelihood of continued growth in the number of registered diabetes patients in the East of England. However, other factors such as dietary information, workload per GP practice, lifestyle (Excess weight, Physical activity, Smoking, Alcohol consumption etc.,), work-life balance, medical history, gender identity, cultural and contextual factors (such as employment and geography), and laboratory test results, etc., need to be considered in future research. A potential future research direction entails the collection of more accurate data to enhance the performance of regression models, thereby facilitating more accurate projections for future outcomes. Additionally, there is an opportunity to broaden the scope of analysis to other regions within the UK, thereby contributing to a more comprehensive understanding of diabetes prevalence. Furthermore, future research involves forecasting shifts in demographics and disease prevalence, and their potential implications for changes in the healthcare workforce. Through these projections, it becomes possible to discern the pressing requirements and potential consequences for regional healthcare workforce demand. This, in turn, allows for the identification of current and forthcoming skill gaps within the workforce and the spatial mapping of these deficiencies.

## Data Availability

The datasets used and/or analysed during the current study are available from the corresponding author on reasonable request.
